# The complete mitochondrial genome of the far Eastern myotis: *Myotis bombinus* Thomas, 1906 in mainland of Korea (Chiroptera, Vespertilionidae)

**DOI:** 10.1080/23802359.2021.1875911

**Published:** 2021-02-17

**Authors:** Byeori Kim, Yoonhyuk Bae, Jungmo Lee, Jongsun Park, Yu-Seong Choi, Heungjin Ryu, Sun-Sook Kim

**Affiliations:** a Division of Ecological Information, National Institute of Ecology, Seocheon, Republic of Korea; b InfoBoss Inc., Seoul, Republic of Korea; c InfoBoss Research Center, Seoul, Republic of Korea; d National Migratory Bird Center, National Institute of Biological Resources, Incheon, Republic of Korea; e School of Life Sciences, Ulsan National Institute of Science and Technology, Ulsan, Republic of Korea

**Keywords:** Mitochondrial genome, *Myotis bombinus*, Chiroptera, Vespertilionidae, South Korea

## Abstract

We have determined the second mitochondrial genome of *Myotis bombinus* Thomas, 1906 in mainland of Korea. The circular mitogenome of *M. bombinus* is 17,035 bp long which is slightly shorter than that of the previous mitogenome of *M. bombinus*. It includes 13 protein-coding genes (PCGs), two ribosomal RNA genes, and 22 transfer RNAs. The base composition was AT-biased (66.1%). Fifty single nucleotide polymorphisms and 14 insertions were identified between two mitogenomes of *M. bombinus*. Phylogenetic trees show that both *M. bominus* mitogenomes are clustered in one clade.

*Myotis bombinus* Thomas, 1906 was distributed in Russia, China, Mongolia, Japan, and Korea (Jargalsaikhan 2016). Especially, *M. bombinus* inhabits whole Korean peninsula including Jejudo island (Won and Smith 1999; Park et al. 2015; Jo et al. 2018). Most typical habitats of *M. bombinus* are forest, cave, and subterranean habitats (Funakoshi 1991; Kim S-S et al. 2014); however, *M. bombinus* became near threatened (NT) species in the world because of loss of these habitats world-widely (Fukui et al. 2019), requiring intensive researches of this species in various ways.

We completed the mitogenome of *M. bombinus*, collected at the Jeombong Mountain, located in Jindong-myeon, Inje-gun, Gangwon-do, Republic of Korea (38°2′32.12′′N, 128°28′23.77′′E). DNA was extracted using DNeasy Blood & Tissue Kit (QIAGEN, Hilden, Germany). Raw sequences from Illumina NovaSeq6000 (Macrogen Inc., Seoul, Korea) were filtered by Trimmomatic v0.33 (Bolger et al. 2014) and *de novo* assembled by Velvet v1.2.10 (Zerbino and Birney 2008), SOAPGapCloser v1.12 (Zhao et al. 2011), BWA v0.7.17 (Li 2013), and SAMtools v1.9 (Li et al. 2009) under the environment of Genome Information System (GeIS; http://geis.infoboss.co.kr/; Park et al., in preparation). Geneious R11 v11.1.5 (Biomatters Ltd, Auckland, New Zealand) was used to annotate our mitogenome based on *M. bombinus* mitogenome (NC_029342; Kim Y-K et al. 2017). DNA sample and tissues of *M. bombinus* were deposited in InfoBoss Cyber Herbarium (IN; Kim S-S; Voucher numbers are IBS-00019 and IB-40004).

*M. bombinus* mitogenome (GenBank accession is MT985383) is 17,035 bp, slightly shorter than that of the previous sequenced mitogenome of *M. bombinus* (NC_029342; 17,128 bp; Kim Y-K et al. 2017). It contains 13 protein-coding genes (PCGs), 22 transfer RNAs, and two ribosomal RNAs. Its AT ratio is 64.7%.

Based on pair-wise alignment of two *M. bombinus* mitogenomes, 50 single nucleotide polymorphisms (SNPs) and 14 insertions of which length was 95 bp in total were found against *M. bombinus* mitogenome isolated in Jejudo island (NC_029342). Twenty out of the 50 SNPs were located in PCGs, displaying that three were non-synonymous SNPs in *ND2* and *COX1* and 17 out of 50 SNPs were synonymous SNPs in eight PCGs. Number of polymorphic sites of four *Myotis petax* mitogenomes (Hwang et al. 2016) is 51, which is almost similar to number of SNPs of *M. bombinus*. In addition, the four samples of *M. petax* are also isolated from Korean peninsula (Hwang et al. 2016), indicating that genetic diversity of *M. bombinus* mitogenomes in Korea is similar to that of *M. petax*.

We inferred phylogenetic relationship of eleven *Myotis* and one *Plecotus* mitogenomes, based on trimmed alignment of mitogenome sequences by MAFFT v7.450 (Katoh and Standley 2013). Bootstrapped neighbor-joining, maximum-likelihood, and Bayesian Inference phylogenetic trees with MEGA X (Kumar et al. 2018) and MrBayes v3.2.7a (Huelsenbeck and Ronquist 2001), respectively, were constructed. Phylogenetic trees display that two *M. bombinus* mitogenomes are clustered in one clade (Figure 1). Taken together, genetic diversity of *M. bombinus* in Korean peninsula can be estimated, especially for genetic difference between Korean peninsula and Jejudo island.

**Figure 1.**
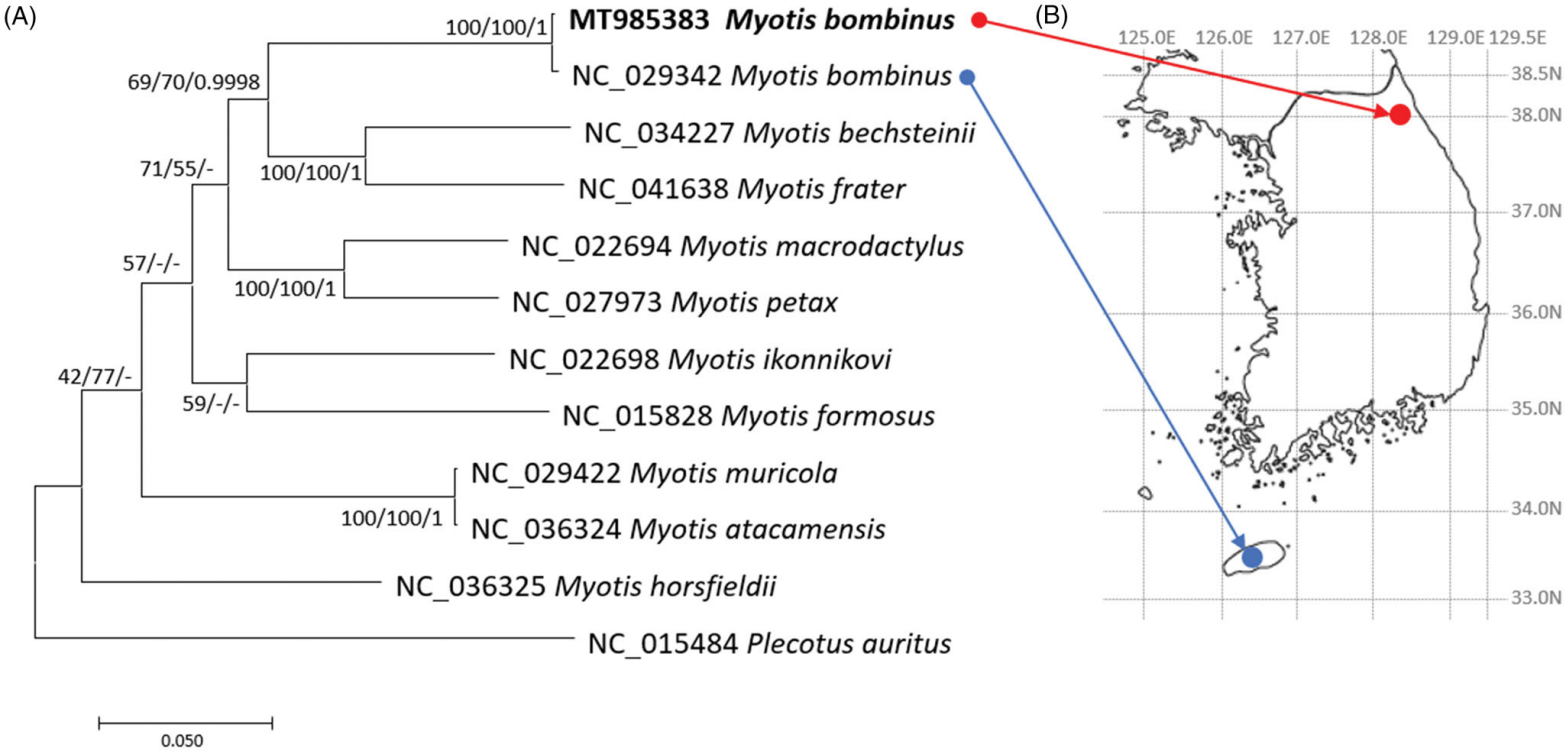
(A) Maximum-likelihood (1,000 bootstrap repeats), neighbor-joining (10,000 bootstrap repeats), and Bayesian Inference (1,100,000 generations) phylogenetic trees of 11 *Myotis* mitochondrial genomes and one *Plecotus* mitogenomes: two *M. bombinus*, *M. bechsteinii*, *M. frater*, *M. macrodactylus*, *M. petax*, *M. ikonnikovi*, *M. horsfieldii*, *M. muricola*, *M. atacamensis*, *M. formosus*, and *P. auratus*. Phylogenetic tree was drawn based on maximum-likelihood tree. The numbers above branches indicate bootstrap support values of maximum-likelihood and neighbor-joining phylogenetic trees and posterior probability value of Bayesian Inference tree, respectively. (B) displays geographical location of two *M. bombinus* samples: red circle indicates the sample used in this study and blue circle means the sample used in the previous study (NC_029342; Kim et al. 2017).

## Data Availability

Mitochondrial genome sequence can be accessed *via* accession number MT985383 in NCBI GenBank.
